# Barriers and facilitators to vaccination for COVID-19, pertussis, and influenza during pregnancy: Protocol for an umbrella review

**DOI:** 10.1371/journal.pone.0275105

**Published:** 2022-09-26

**Authors:** Bethany Nichol, Valentina Simonetti, Jemma McCready, Mary Steen, John Unsworth, Marco Tomietto

**Affiliations:** 1 Department of Social Work, Education and Community Wellbeing, Faculty of Health and Life Sciences, Northumbria University, Newcastle upon Tyne, United Kingdom; 2 Department of Biomedical Science and Human Oncology, University of Bari "Aldo Moro", Bari, Italy; 3 Department of Nursing, Midwifery and Health, Faculty of Health and Life Sciences, Northumbria University, Newcastle upon Tyne, United Kingdom; 4 Research Unit of Nursing Science and Health Management, University of Oulu, Oulu, Finland; 5 Universita’ degli Studi di Bari “Aldo Moro”, Bari, Italy; Kyung Hee University School of Medicine, REPUBLIC OF KOREA

## Abstract

Pregnant women are particularly vulnerable to infection. Furthermore, infection from pertussis, influenza and COVID-19 increases the likelihood of adverse consequences to the mother and developing baby such as stillbirth, ICU admission, and pre-term caesarean birth. Increased rates of transmission and risk of adverse consequences from infection justifies the provision of national maternal vaccination programmes. Additionally, maternal vaccination helps protect the infant until they are able to receive their own vaccinations; a time when they are most at risk of mortality from influenza and pertussis. Vaccination during pregnancy has been repeatedly demonstrated as safe and effective in reducing harm, although rates of uptake remain low compared to the general population. The current protocol describes the methodology for an umbrella review aiming to explore the barriers and facilitators of vaccination during pregnancy for pertussis, influenza, and COVID-19. Systematic reviews that investigate the barriers and facilitators of at least one of either pertussis, influenza, or COVID-19 will be included in this review. Multiple databases will be searched, and included reviews assessed for quality (using the Joanna Briggs Institute (JBI) quality assessment for systematic reviews) and degree of overlap of included primary studies. Included reviews will be analysed according to the WHO SAGE model of determinants of vaccine hesitancy and separated by whether these explore influenza and pertussis, or COVID-19. The outcomes of this review will help inform the development of interventions to increase uptake of vaccination during pregnancy, and on whether interventions need to be tailored depending on the infectious disease. The key findings will identify the specific barriers and facilitators of vaccination hesitancy by considering contextual influences (e.g. sociodemographic variables), individual/social group influences (e.g. trust in the institutions), and vaccine-specific issues (e.g. safety and recommendations).

## Introduction and rationale

Women during pregnancy are particularly vulnerable to infectious agents when compared with non-pregnant women of the same reproductive age, because of immunological changes that may alter their susceptibility to and severity of infectious diseases [1]. Subsequently, recent epidemics such as COVID-19, H1N1 flu, and Pertussis, have generated high rates of maternal and foetal morbidity and mortality [2–4].

Maternal vaccination programmes remain effective [5–12], and safe strategies [9, 12–18] for a number of infectious agents. The beneficial effects of these programmes may not be limited to the mother and developing baby, but also have potential long-term benefits for the new-born infant [19]. For example, infants under the age of six months (for pertussis and influenza) and five years (for COVID-19) are unable to produce adult-like immune responses [20], yet they do not receive vaccination, despite being at their most vulnerable for mortality from pertussis [21] and influenza [22, 23]. Therefore, maternal vaccination ensures the infant is protected before they receive vaccinations of their own.

In high-income countries, the World Health Organization (WHO) recommends vaccination for pertussis, influenza [24] and COVID-19 [25] to all pregnant and lactating women, in order to prevent adverse consequences, such as an increased risk of stillbirth and Intensive Care Unit (ICU) admission after birth for COVID-19 [26, 27], severe respiratory illness during COVID-19 and/or influenza, and pneumonia for all mentioned pathologies [28]. Additionally, vaccination protects mothers from exacerbation of symptoms with the possibility of hospitalization, and reduces the risk of pre-term and caesarean birth [27, 29, 30].

Unfortunately, achieving a good vaccination coverage among pregnant and lactating women remains a global challenge [28] both for influenza and pertussis vaccines [31–34]. During the COVID-19 outbreak, a disruption in routine immunization programmes around the world occurred and resulted in a subsequent decline in vaccine uptake [35]. Recent evidence reported that COVID-19 vaccine coverage and/or acceptance was lower among pregnant women when compared to non-pregnant women [36–38], and even lower for pregnant women living in the most deprived areas [36].

The negative consequences linked to low immunization not only impacted on the healthcare systems of Countries worldwide, but also on their economies. Thus, considering critical factors for vaccine success [39] and promoting awareness of vaccination campaigns, particularly if specifically tailored by population groups, should be a cost-effective solution to increase coverage rates [40] and raise public confidence in usage.

Among barriers to gain a high vaccination uptake we recognize “vaccine hesitancy”, a behaviour influenced by different issues: *(I) confidence* (distrust in the vaccine or provider), *(II) complacency* (no perceived need for a vaccine, vaccine not valued) and *(III) convenience* (access issues) [41, 42], which places the individual on a continuum between indecision and anti-vaccination [43, 44]. Vaccine-hesitant individuals may accept all vaccines but remain concerned about the safety of vaccination, some may refuse or delay some vaccines but accept others, some may refuse all vaccines because of a lack of trust in the government or in the healthcare system, and others may be suspicious of pharmaceutical companies profiteering from sales [45–47].

The WHO-SAGE “Model on determinants of vaccine hesitancy” [42] classifies individual behaviour about vaccination into three domains: *(I) Contextual influences* (historic, socio-cultural, environmental, health system/institutional, economic or political factors); *(II) Individual and group influences* (personal perception of the vaccine or influences of the social/peer environment); and *(III) Vaccine and vaccination-specific issues* (related to the characteristics of the vaccine or the vaccination process).

Recent literature suggests a difference between determinants of COVID-19 vaccine hesitancy and other infectious diseases. For example, concerning influenza and pertussis vaccination, the main reasons for hesitancy among pregnant women were: limited knowledge, poor awareness of the advantages of getting vaccinated and available sites for vaccination, fear of the vaccine contents and side effects, doubt on vaccine efficacy, and healthcare workers not recommending vaccinations [28]. Regarding COVID-19 outbreak, the evolving pandemic and vaccines, the newer variant, evolving virus strains, lack of information/misinformation circulating on social media, fake news, anti-vaccine movements [48], development of vaccines in a fast manner, and political intervention [49] may have contributed to a negative perception towards the efficacy and safety of new vaccines [50]. Additionally, individual factors including different backgrounds, experiences, and beliefs related to COVID-19, socio-economic status, education, pregnancy, employment, and trust in the government may further contribute to vaccine hesitancy [50, 51].

Understanding the reasons for vaccination hesitancy and/or low coverage in pregnancy (which can be linked to the individual woman, the vaccinator, or policies or structural factors) is a prerequisite for addressing concerns [39, 52] and implementing interventions to increase vaccination uptake for flu, pertussis and COVID vaccines.

Considering the factors influencing the uptake of vaccinations for specific sub-populations such as pregnant women is an urgent need that must be met in order to direct public health vaccination campaign to prevent COVID-19, pertussis and influenza diseases.

This umbrella review (or systematic review of systematic reviews) will examine factors (barriers and facilitators) and the possible domains of intervention (contextual, individual/social and vaccine-specific) associated with vaccination hesitancy among pregnant women, to gain a full comprehension of the phenomena. Given the large number of primary studies and reviews on this topic in the last 11 years, an umbrella review, or review of reviews, is needed to achieve a clear overview of the evidence available and to address future research and public health policies.

### Protocol aim and research questions

This umbrella review aims to identify and synthesise currently available knowledge to determine the barriers and facilitators to vaccination uptake among pregnant women.

*Two questions will be addressed*:

1. What are the barriers and facilitators to the uptake of vaccinations to prevent COVID-19, pertussis and influenza amongst women during pregnancy?
2. Which are the most relevant domains (contextual, individual/social and vaccine-specific) of vaccine hesitancy?

## Materials and methods

This protocol was registered in the International prospective register of systematic review PROSPERO, available at (*blinded for referees*): and designed by following the Joanna Briggs Institute guidelines for the conduct and preparation of umbrella reviews [53] and the Preferred Reporting Items for Systematic review and Meta-Analysis Protocols (PRISMA-P) guidelines for the reporting of systematic review protocols [54].

To ascertain that there are no existing umbrella reviews on this topic, a preliminary search of three systematic review registries was first undertaken (the International Prospective Register of Systematic Review [PROSPERO], Joanna Briggs Institute Systematic Review Register, and Open Science Framework Registries).

### Inclusion criteria

Systematic reviews and meta-analyses evaluating barriers, facilitators or factors associated with COVID-19, pertussis, or influenza vaccinations among women during pregnancy will be included.

#### Participants

Any pregnant adult over 18 years of age will be included. Studies with partially overlapping age ranges will also be included (e.g., 16+). For example, whilst reviews only focusing on determinants of vaccine hesitancy in adolescents would be excluded, reviews that may include some participants over 16 would be included. The full sample must be pregnant or have been pregnant within the last two years.

#### Outcomes

As the primary outcome, studies establishing the barriers and facilitators to vaccine hesitancy, using the ‘Model of determinants of vaccine hesitancy’ by Wilson et al. [28] as a framework will be included. Reviews that only measure uptake or hesitancy rates will be excluded.

As secondary outcomes, review or meta-analysis discussing interactions between barriers and facilitators and reported descriptive data on vaccine hesitancy scores, or uptake will be eligible for inclusion.

#### Measures of effect

Included quantitative studies may either measure the frequency and proportion of identified barriers and facilitators by participants, or the prediction of named barriers and facilitators in predicting vaccine uptake. Thus, measures of effect may take the form of frequencies, percentages, or odds ratios. Reviews may report the frequency of studies that have explored certain barriers and facilitators, and what proportion of these yielded a significant or non-significant result.

Any reviews including meta-analysis will allow for a formal measure of effect in the form of heterogeneity statistics, pooled estimates, and confidence intervals.

For reviews that performed a meta-analysis on descriptive statistics on uptake and /or vaccine hesitancy scores or rates, these will be analysed as additional outcomes, again in the form of heterogeneity statistics, pooled estimates, and confidence intervals.

#### Types of studies

Systematic reviews with or without meta-analysis will be included. Reviews including only or mostly quantitative studies, from all countries and all study settings, will be included.

Primary studies will be excluded, although may be used for forward citation searching for reviews.

### Search strategy

Databases will be searched from 2009 to 22^nd^ April 2022 (when the search will be undertaken). Reviews from 2009 onwards will be included to capture the influence of the influenza pandemic, during which pregnant women were disproportionately affected [55], the pandemic influenza vaccine was made publicly available [56], and the first RCT demonstrating the effectiveness of seasonal influenza vaccination during pregnancy was published [57]. The following databases will be searched: Consumer Health Database, Health & Medical Collection, Healthcare Administration Database, MEDLINE^®^, Nursing & Allied Health, Database, Psychology Database, and Public Health Database, EPISTEMONIKOS, and PsycARTICLES. Searches will be restricted to peer-reviewed reviews.

Scoping searches have been completed prior to the review search and the search strategy was discussed with a University librarian and checked against the PRESS statement [58]. Alerts will be created for the search so that it is kept as up to date as possible before publication. These will be terminated in July 2022. Further sources to identify reviews will be forward and backward citation searching of included reviews, and through colleagues and other academics. Primary studies, commentaries, opinion pieces will be excluded. Only reviews published in English will be included.

References of included studies will be searched for additional systematic reviews.

The search will be run again in the synthesis stage to identify any relevant reviews published since the initial search.

The search strategy will consider the following keywords as displayed in Table 1.

**Table 1:** Search strategy.

Vaccination	Hesitancy	For: COVID and Influenza	In pregnant women
vaccin* OR immunis* OR immuniz* OR incoculat*	Anxiet* OR doubt* OR trust* OR intent* OR dilemma* OR attitude* OR distrust OR mistrust OR controvers* OR objector* OR awareness OR dropout* OR Perception* OR misconception* OR uptake OR behavi*r OR exemption* OR refus* OR misinform* OR barrier* OR belief* OR fear* OR reject* OR oppos* OR choice* OR criticis* OR hesitanc* OR rumo*r OR delay OR accept* OR concern* OR knowledge OR confiden* OR decision OR anti-vaccin* OR predict* OR factors OR failure OR affect OR reason* OR utilis* OR utiliz* OR worry OR facilitate* OR enable* OR implement* OR frequency OR cause* OR willing* OR perspective* OR determine* OR react* OR indecision OR reluct*	Influenza OR H1N1 OR H5N1 OR flu OR TIV OR IIV3 OR IIV4 OR COVID OR COVID19 OR "SARS-CoV-2" OR "SARS-CoV2" OR SARSCoV2 OR "SARSCoV-2" OR "SARS coronavirus 2" OR "2019 nCoV" OR "2019nCoV" OR "2019-novel CoV" OR "nCov 2019" OR "nCov 19" OR "severe acute respiratory syndrome coronavirus 2" OR "novel coronavirus disease" OR "novel corona virus disease" OR "corona virus disease 2019" OR "coronavirus disease 2019" OR "novel coronavirus pneumonia" OR "novel corona virus pneumonia" OR "severe acute respiratory syndrome coronavirus 2" OR ‘whooping cough’ OR ‘pertussis’ OR ‘tdap’	maternal OR antenatal OR prenatal OR pregnan* OR perinatal

(See Supplemental material for a detailed search strategy, available at: (*PROSPERO protocol blinded for referees*).

### Study selection

Search results will be exported via a RIS file and uploaded onto Rayyan, a free web and mobile app, that helps expedite the initial screening of abstracts and titles using a process of semi-automation while incorporating a high level of usability [59].

One reviewer (*initials blinded for referees*) will screen all reviews for title and abstract against the inclusion criteria, followed by the remaining articles based on full text. A second member of the research team (*initials blinded for referees*) will independently screen the first 10% of articles at both stages, sorted alphabetically by author. Decisions will be made and logged via Rayyan. Any disagreements will be discussed and resolved when both reviewers reach a consensus.

Data will be extracted using guidance from Cochrane [60] (Pollock et al., 2020) As with article screening, a second member of the research team will extract data from the first 10% of included studies and any disagreements will be discussed until a consensus is reached.

The following data will be extracted:

(i) General (review information, funding source, contact details, conflicts of interest); (ii) Review information (aim of the review, search strategy, exclusion criteria, any frameworks or theories that were used); (iii) Primary included studies information (% of quantitative studies, year, country and population); (iv) Results (information on the barriers and facilitators discussed, any significant or non-significant findings, interactions reported, results of meta-analysis if applicable); (v) Additional information (conclusions of authors, limitations, quality score, any correspondence with authors).

Where there is missing or unclear reporting, study authors will be contacted where possible.

The data extraction form will be completed for each review via Microsoft Excel (2018).

### Assessment of methodological quality

The Joanna Briggs Institute Critical Appraisal Checklist for Systematic Reviews and Research Syntheses [61] will be used to assess study quality. The checklist covers clarity of the review question, and the appropriateness of the search strategy, inclusion criteria, quality assessment, methods for data synthesis, and conclusions made. Additionally, there are question prompts to ensure that the quality assessment used was adequate.

Quality of the studies will be considered when synthesising the results. If study quality is judged to be extremely low, it will be excluded from the review. Again, a second member of the research team (*blinded for reviewers*) will assess 10% of studies for quality. Cohen’s kappa statistic (*k*) will be calculated to ascertain the degree of agreement between reviewers [62]. Where there are disagreements, results will be discussed until a consensus is reached and the study is included or excluded.

For quality of individual studies, quality appraisal by the review will be used. Where two reviews include the same primary study, the appraisal by review of the higher quality will be used. If the reviews are both of the same quality, the review using the most rigorous quality assessment tool will be used. A table will be created to note the different quality appraisal tools used by reviews and their quality assessment for included primary studies.

### Strategy for data synthesis

Table mapping the primary studies contained within included systematic reviews will decipher the degree of overlap between reviews. ‘Corrected cover area’ (CCA) will be calculated using the formula provided by Pieper et al. [63], which divides the number of repeated primary studies across the included reviews by the number of rows (primary studies) and columns (reviews) of the overlap table multiplied together minus the number of rows. This measure is favourable as it is sensitive to primary studies repeated in more than one included review and gives a percentage coverage between 0–100%. As per the guidance of Pieper, the CCA will be reported and recognised as a limitation, if necessary, although no reviews will be removed from analysis based on this statistic.

The remaining data contained within the systematic reviews and meta-analyses will be collated, and a descriptive numerical summary and a thematic analysis will be conducted. The numerical summary will describe the characteristics of the included studies in table format. This table will include the review’s aim, search strategy, inclusion criteria, outcomes, population, and conclusions made by the authors.

A narrative summary of the key determinants will be provided, which will describe in detail the barriers and facilitators of vaccine hesitancy towards the COVID-19, influenza, and pertussis vaccines.

Outcome data extracted from the reviews will be analysed separately for COVID-19, and pertussis and influenza, and findings from these will be compared and contrasted in a final narrative summary.

Risk of bias assessments for primary studies made by the included reviews will be presented in a table format, and a narrative summary will discuss their disagreements in terms of the quality appraisal of the reviews themselves.

All the tables and the supplementary materials will be available in an open access format in public repositories. The template spreadsheet for data extraction is available on Open Science Framework (https://osf.io/g94nu/?view_only=42ea0601bbe246a198d35c6dacfcb56e), where the completed table will be uploaded too.

## Discussion and implications

This review will inform the development of interventions to increase uptake of vaccination during pregnancy, and on whether interventions need to be tailored depending on the infectious disease. The key findings will identify the specific barriers and facilitators of vaccination hesitancy by considering contextual influences (e.g. sociodemographic variables), individual/social group influences (e.g. trust in the institutions), and vaccine-specific issues (e.g. safety and recommendations), according to the WHO SAGE international model of determinants of vaccine hesitancy [42]. This umbrella review will summarize the evidence of the last 13 years on the topic and it will provide useful insights for future research for promoting vaccination uptake during pregnancy. The identification of the main determinants of vaccine hesitancy during pregnancy will also inform policymakers on the specific areas to focus in order to tailor the vaccination campaigns at the public health level. Tailored interventions in the maternal services could be designed, based in this review’s findings, to promote maternal health and immunization. The findings will also support the identification of the specific level of intervention from the individual level (e.g. specific age-categories) to the community (e.g. specific ethnic groups) and organisational level (e.g. specific healthcare workers (HCWs) or services which could recommend/promote the vaccination; educational interventions addressed to HCWs to engage them in the vaccination process).

### Study limitations

The umbrella review approach presents intrinsic limitations that should be discussed.

While it enables the researchers to perform a global assessment of a broad topic in a systematic manner, results could be limited to the strict criteria of included studies in each systematic review. Furthermore, whilst EPSISTEMONIKOS was searched to access reviews specifically, important databases such as Cochrane were not accessed, potentially limiting the comprehensiveness of the current umbrella review. Lastly, given the extensive timespan of our umbrella review, we expect a large/moderate degree of overlap between primary studies within the included reviews, inflating the impact of these duplicated primary studies [64]. Whilst this will be addressed by showing all duplicated primary studies in table format and calculating CCA as described, this only serves to outline potential overlap as a limitation rather than removing its impact.

## Conclusion

This protocol addresses a major public health issue in a particularly vulnerable population. Despite the wide number of systematic reviews on this topic, there is no recent summary of the findings related to vaccination facilitators and barriers in the pregnant women population. This protocol fills this gap, and its outcomes will inform the policymakers about the most effective public health strategies to promote vaccination uptake among pregnant women.

## Supporting information

S1 Checklist PRISMA-P (Preferred Reporting Items for Systematic review and Meta-Analysis Protocols) 2015 checklist: Recommended items to address in a systematic review protocol*. (DOCX)
